# Management and Outcomes of ANCA-Associated Vasculitis at a Tertiary Healthcare Facility

**DOI:** 10.1155/2022/4808806

**Published:** 2022-02-11

**Authors:** Nabeehah Moollan, Adeel Rafi Ahmed, Mark Denton

**Affiliations:** ^1^Nephrology Department, Beaumont Hospital, Dublin, Ireland; ^2^University of South Wales, CF37 1DL, Pontypridd, UK

## Abstract

**Results:**

Thirty-six patients were included in the final study. Cyclophosphamide was used in 24 patients (66.7%) and, comparatively, rituximab in 7 patients (19.4%) for induction. Seven patients (19.4%) had a documented relapse, and six patients (85.7%) had rituximab as induction therapy for relapse. The majority of patients were on azathioprine (61.1%, 57.1% relapse population) as maintenance therapy. Progression to ESRD occurred in 11 (30.6%), death in 4 (11.1%), established CKD in 15 (41.7%), and preservation of renal function in 6 (16.7%) patients by the end of the follow-up period.

**Conclusions:**

While cyclophosphamide remains the choice of induction immunosuppression therapy, we favour rituximab as an induction agent in the relapse of AAV. Despite aggressive immunosuppression therapy, the incidence of ESRD and death remains high in these patients.

## 1. Introduction

Antineutrophil cytoplasmic antibody- (ANCA-) associated vasculitis (AAV) is a group of disorders including granulomatosis with polyangiitis (GPA), eosinophilic granulomatosis with polyangiitis (EGPA), and microscopic polyangiitis (MPA), which are characterised by systemic necrotising inflammation in small- and medium-size vessels leading to end-organ damage [1, 2].

The overall annual incidence of AAV is estimated to be around 0.4–24 per million population per year [3]. In the United Kingdom, the incidence and prevalence rate of GPA are 9.7 and 62.9 per million population compared to 13 and 218 per million population in the United States of America [4, 5]. The prevalence of MPA approximately ranges from 9 to 94 per million population [3]. The prevalence of AAV is more common in people of European ancestry [5]. In Southern Europe and Asia, MPA is more prevalent, while in Northern Europe, GPA is more common [6]. The incidence increases with age, with a peak observed in the 60–70 years old category [7].

Immunosuppression agents are used to induce remission and as a long-term maintenance treatment. In life-threatening or organ-threatening AAV, cyclophosphamide or rituximab with glucocorticoid is recommended as induction agents. The addition of plasmapheresis (PLEX) can be considered for rapidly progressive renal failure or severe diffuse pulmonary haemorrhage [2–4]. Azathioprine, rituximab, or methotrexate, with glucocorticoid tapering, is recommended maintenance therapies [8]. Cyclophosphamide is the conventional induction agent used by nephrologists in AAV. Rituximab, an anti-CD20 monoclonal antibody, has shown noninferiority to cyclophosphamide and is increasingly being used for induction and remission maintenance in AAV [9].

Renal involvement is one of the main predictors of mortality and morbidity in AAV, with approximately 20–30% of patients with renal involvement, progressing to ESRD [10, 11]. One of the main predictors of poor renal outcome is elevated serum creatinine at the time of diagnosis [11].

Histopathological evidence of AAV on renal biopsies, such as necrotizing, and crescentic glomerulonephritis without significant immune complex deposition (pauci-immune glomerulonephritis), can provide a definite diagnosis and may predict the renal prognosis in AAV [7]. Thus, it remains the gold standard for diagnosing renal involvement in AAV [2].

AAV is a rare disorder with limited data available on its management and outcomes [3]. Therefore, we aim to assess the management and outcomes of organ-threatening AAV at our tertiary-care renal facility.

## 2. Methods

Study design: this was a retrospective cohort study. We included patients with a documented diagnosis of ANCA-positive vasculitis between the 1^st^ of January 2012 and the 31^st^ of December 2017, with a follow-up period until the 31st of December 2019. Data were collected retrospectively, using the electronic renal database (eMED), which included data on outpatient encounters, inpatient care, patient laboratory, and histology results, at our nephrology department in Beaumont Hospital, Dublin.

The inclusion criteria were those with a clinical diagnosis of AAV consistent with the Chapel Hill consensus definition, positive serology, i.e., antiproteinase (anti-PR3) or antimyeloperoxidase (antiMPO) antibody positivity, renal biopsy showing pauci-immune necrotising glomerulonephritis, or active urine sediment characterised by glomerular haematuria or red cell casts and proteinuria, or clinical features consistent with AAV. The exclusion criteria were patients with a diagnosis of vasculitis other than granulomatosis with polyangiitis or microscopic polyangiitis or renal histological features suggestive of other types of glomerulonephritis such as linear IgG deposition on immunofluorescence, age less than 16 years, and patients with incomplete records.

Patient characteristics: patient characteristics, including age, gender, comorbidities, along with a retrospective calculation of Birmingham Vasculitis Activity Score (BVAS) and organ systems involved, creatinine levels, and anti-PR3/anti-MPO titres, at presentation, were recorded.

Outcomes and definitions: using a standardised data collection form, we recorded the number of patients who had a renal biopsy, renal biopsy light microscopy, and immunofluorescence results, initial induction treatment, and maintenance treatment, along with relapse induction and maintenance therapies. Disease relapse was defined as the intensification of therapy due to worsening of disease activity as evidenced by clinical and/or biochemical findings.

At the end of the follow-up period, outcomes were divided into end-stage renal disease (ESRD) or death, established chronic kidney disease (CKD), and preservation of renal function. ESRD was defined as those requiring renal replacement therapy, CKD was defined as an eGFR of less than 60 ml/min/1.73 m^2^, and preservation of renal function as eGFR more than 60 ml/min/1.73 m^2^, at the end of the follow-up period.

Statistical Analysis: summary statistics of continuous variables were given by mean and standard deviation (SD), or median and interquartile range (IQR, 25^th^ and 75^th^ percentile), as appropriate. Categorical variables were summarised by frequencies (percentages). The chi-square test was used to look for statistical significance between a categorical variable, and a *p* value of <0.05 was considered significant.

## 3. Results

### 3.1. Participants

Forty-two patients with PR3-ANCA- and MPO-ANCA-associated vasculitis were identified between 2012 and 2017 with a median follow-up period of 6 years. Six patients were excluded in the final analysis due to histological features suggestive of other types of glomerulonephritis on renal biopsy, unclear diagnosis, or incomplete records.

### 3.2. Patient Characteristics

Data of thirty-six patients were included in the final study. The patient baseline characteristics on presentation are listed in Table 1. The median age at presentation was 63.5 years. The majority were male (66.7%). The mean BVAS score was 15. All had severe renal involvement, and the other organ systems involved included cutaneous (15 patients), respiratory (12 patients), Ear, Nose, and Throat (ENT) (9 patients), joint involvement (4 patients), neurology (3 patients), and gastrointestinal (2 patients). The organ systems involved are listed in Table 2. The median creatinine was 301 *µ*mol/L. The median anti-PR3/anti-MPO titre was 28 IU/ml. Twenty patients (55.6%) had anti-MPO antibody positivity, compared to 16 patients (44.4%) with anti-PR3 antibody positivity.

## 4. Outcomes

### 4.1. Renal Biopsy Performed

Thirty-two patients (94.1%) had a documented renal biopsy. Of the four remaining patients, one patient was documented as unsuitable for biopsy due to comorbidities, another patient had a resolved AKI on transfer to Beaumont Hospital, and as such was not biopsied, and the other two patients were transferred to Beaumont Hospital with no documented biopsy on our system.

All the performed biopsies were assessed with light microscopy and immunofluorescence, and showed pauci-immune necrotising and crescentic glomerulonephritis.

### 4.2. Induction and Maintenance Therapy

Twenty-four patients (66.7%) had cyclophosphamide, and seven patients (19.4%) had rituximab for induction. Four patients (11.1%) had azathioprine as an induction; this was due to mild symptoms and or comorbidities. One patient (2.8%) had a combination of low-dose cyclophosphamide and rituximab. The majority of patients were on azathioprine (61.1%) as maintenance therapy.

### 4.3. Relapse

Seven patients (19.4%) had a documented relapse during the study period. The mean time to relapse was 3.4 years, and the median time to relapse was 3 years. Of these, 4 (57.1%) were anti-MPO antibody positive and 3 (42.9%) were anti-PR3 antibody positive. Six patients (85.7% of those who relapsed) had rituximab as induction therapy for relapse, with one patient who had nonrenal involvement of his relapse receiving steroids alone. Four patients (57.1%) were commenced on azathioprine as maintenance therapy, with two (28.6%) being maintained on rituximab. The summary of induction and maintenance treatment is listed in Table 3.

### 4.4. Outcome: Progression to ESRD or Death

The median follow-up period was 6 years. Progression to ESRD occurred in 11 patients (30.6%) and death in 4 patients (11.1%) by the end of the follow-up period. The mean time to ESRD was 1.1 years, and the median time to ESRD was <1 year.

Ten patients (66.7%) with ESRD or death had induction therapy with cyclophosphamide. Two (13.3%) were induced with rituximab and continued on steroids. Three (20%) were started on azathioprine due to comorbidities.

Of those induced with cyclophosphamide, the majority (6 patients) were commenced on azathioprine for maintenance, one was commenced on mycophenolate mofetil (MMF), and corticosteroids alone were used in three patients.

Of those induced with cyclophosphamide, 41.7% progressed to ESRD or death compared to 28.6% of those induced with rituximab. This was not statistically significant (*p*=0.53). 40.9% of those on azathioprine and 66.7% of those on corticosteroids alone as maintenance progressed to ESRD or death.

The 11 patients with ESRD had various renal replacement therapy modalities, with 6 started on haemodialysis, 1 peritoneal dialysis, and 4 renal transplants.

### 4.5. Outcome: CKD and Preservation of Renal Function

Fifteen patients (41.7%) had established CKD, and 6 patients (16.7%) had preservation of renal function by the end of the follow-up period.

Eleven patients (73.3%) with established CKD had cyclophosphamide as induction, two (13.3%) had rituximab, and one (6.7%) had cyclophosphamide and rituximab, with one (6.7%) being induced with azathioprine. Three patients (50%) with preservation of renal function were induced with cyclophosphamide and three (50%) with rituximab. The majority of patients with CKD were maintained on azathioprine (11 patients, 73.3%), with 3 patients (50%) with preservation of renal function being maintained on rituximab and 2 (33.3%) on azathioprine.

Of those induced with cyclophosphamide, 45.8% had established CKD, compared to 28.6% induced with rituximab. 42.9% induced with rituximab had preservation of renal function compared to 12.5% of those induced with cyclophosphamide. These results are listed in Tables 4 and 5.

## 5. Discussion

Cyclophosphamide remained the choice of induction therapy in AAV with severe renal involvement; however, despite such aggressive immunosuppression, a high proportion of patients developed ESRD (30.6%) and significant mortality (11.1%), which is comparative to the previous data [1, 2]. The mean time to develop ESRD was 13 months from the time of diagnosis of AAV. The majority of the studies have demonstrated that the most important predictor of adverse renal outcome is the elevated serum creatinine at the time of presentation of AAV [12, 13]. In our cohort, the median creatinine at presentation was 301 *µ*mol/L (3.4 mg/dL), indicating significant renal involvement that reflected the high incidence of ESRD within the first 13 months of diagnosis. In a large Dutch study, a similar outcome was noted with the majority of ESRD incidence occurring within the first year of diagnosis of AAV [14].

MPA was relatively more common in our study compared to GPA (Table 1). The UK and Ireland Vasculitis Registry (UKIVAS), the largest registry on AAV in the world, showed MPA involved the kidneys more commonly compared to GPA (86% vs. 55%) even though the overall prevalence of MPA was lower than that of GPA [15]. The patients referred to our tertiary-care facility all had renal involvement; thus, MPA was more common.

Azathioprine was the choice of maintenance therapy which is standard practice as it has shown to be equivalent to cyclophosphamide and superior to mycophenolate mofetil (MMF) in remission maintenance [16, 17]. The use of rituximab for the maintenance of remission in AAV and, particularly, GPA is becoming more common in Europe and the USA; however, robust evidence supporting this practice was not widely available at the time of our study period and only recently has been incorporated into guidelines [15, 18]. Thus, a minority of patients were maintained on rituximab in our cohort (17%) compared to azathioprine (61%).

In our cohort, rituximab was favoured as an induction agent in the relapse of AAV (86%). Cumulative exposure to cyclophosphamide is associated with an increased incidence of malignancy, and recent studies have shown a high remission rate by reinduction with rituximab in AAV [12–14]. In a minority of patients who had significant comorbidities and/or were commenced on haemodialysis, azathioprine or steroids alone were used. Around 19% of patients relapsed, with a median time to relapse being three years. Data suggest around 30–50% of patients with AAV relapse within five years [19]. However, the highest risk of relapse is seen in AAV with anti-PR3 and preserved renal function [19]. Other risk factors for relapse include ANCA positivity at the time of completion of induction therapy, staphylococcus aureus nasal carriage, choice of induction and maintenance therapy, and short duration of maintenance treatment [20].

The PLEX was used in 27.8% of patients, indicating severe renal involvement with or without pulmonary haemorrhage. A similar criterion from the Randomised Trial of Plasma Exchange or High-Dosage Methylprednisolone as Adjunctive Therapy for Severe Renal Vasculitis (MEPEX) study was used when considering PLEX (serum creatinine more than 500 *µ*mol/L) [21]. The results of the Plasma Exchange and Glucocorticoids in Severe ANCA-Associated Vasculitis (PEXIVAS) trial suggest that the use of PLEX does not reduce the incidence of ESRD and death in AAV, which may alter our reliance on its use in the future [22].

The majority of patients in our cohort had residual renal damage and developed CKD (41.1%), which significantly contributes to mortality, morbidity, and healthcare economic burden [16]. The five-year survival for AAV is estimated to be around 75%, with early mortality being attributed to active vasculitis or infection and late mortality being due to infection, cardiovascular disease, and malignancy [1, 21]. In a large randomised control trial, induction with rituximab or cyclophosphamide led to no statistically significant difference in the incidence of ESRD or death, which is similar to our findings [23]. Novel therapeutic agents such as avacopan, a C5a receptor inhibitor and steroid-sparing agent, may contribute to improving long-term outcomes in AAV [24].

Our study limitations include small sample size and single-centre data. As this was a retrospective cohort study, some patients were excluded due to incomplete records. We did not have the electron microscopy renal biopsy results, which could have revealed additional renal diseases. Our study has potential for referral bias, as more severe cases are managed under our service, as it is a large nephrology department in a tertiary-care centre. Despite these limitations, our paper provides clinically important information on this rare disease's management and outcomes.

In conclusion, despite aggressive immunosuppression therapy and improved understanding of the pathogenesis of AAV, the incidence of ESRD and death remains high in these patients. Cyclophosphamide is used more commonly as an inducing agent, while rituximab is utilised more often in relapsing disease in this single-centre experience. Novel therapies are required to reduce morbidity and mortality.

## Figures and Tables

**Table 1:** Baseline demographics and laboratory values at presentation.

Summary following inclusion and exclusion criteria	*N* = 36
Age (years)^a^	63.5 (54.5–71.5)
Gender (male, %)	23 (66.7)
Hypertension (%)	26 (72)
Hyperlipidaemia (%)	8 (22.2)
Ischaemic heart disease (%)	4 (11.1)
Gout (%)	3 (8.3)
Diabetes mellitus (%)	2 (5.6)
BVAS^b^	15 ± 4
Creatinine^a^ (ml/min/1.73 m^2^)	301.5 (169–718.5)
Anti-PR3/antiMPO titre^a^ (IU/ml)	28 (12.5–87.5)
Anti-MPO antibody positive (%)	20 (55.6)
Anti-PR3 antibody positive (%)	16 (44.4)

^a^Median (interquartile range). ^b^Mean ± standard deviation.

**Table 2:** Other organ systems involved.

Organ system	Number of patients
Cutaneous	15
Respiratory	12
Ear, Nose, and Throat (ENT)	9
Joint involvement	4
Neurology	3
Gastrointestinal	2

**Table 3:** Summary of induction and maintenance treatment.

Therapy	Induction, % (*n* = 36)	Maintenance, % (*n* = 36)	Relapse induction, % (*n* = 7)	Relapse maintenance, % (*n* = 7)
Cyclophosphamide	66.7 (24)	0 (0)	0 (0)	0 (0)
Rituximab	19.4 (7)	16.7 (6)	85.7 (6)	28.6 (2)
Cyclophosphamide and rituximab	2.8 (1)	0 (0)	0 (0)	0 (0)
Azathioprine	11.1 (4)	61.1 (22)	0 (0)	57.1 (4)
Corticosteroids alone	0 (0)	16.7 (6)^a^	14.3 (1)	14.3 (1)
Plasmapheresis	27.8 (10)	0 (0)	0 (0)	0 (0)
Mycophenolate mofetil	0 (0)	2.8 (1)	0 (0)	0 (0)
No treatment	0 (0)	2.8 (1)^b^	0 (0)	0 (0)

^a^Due to the commencement of HD or death in 4 (66.7%) of the patients, no information is available on 2 (33.3%) patients. ^b^Not given further immunosuppression due to bowel perforation following cyclophosphamide. However, they remained clinically stable, with no worsening renal function.

**Table 4:** Summary of induction and maintenance treatment: breakdown of patients with ESRD or death, CKD, and preservation of renal function.

Therapy	Induction: ESRD/death, % (*n* = 15)	Maintenance: ESRD/death, % (*n* = 15)	Induction: CKD, % (*n* = 15)	Maintenance: CKD, % (*n* = 15)	Induction: preservation of renal function, % (*n* = 6)	Maintenance: preservation of renal function, % (*n* = 6)
Cyclophosphamide	66.7 (10)	0 (0)	73.3 (11)	0 (0)	50 (3)	0 (0)
Rituximab	13.3 (2)	6.7 (1)	13.3 (2)	13.3 (2)	50 (3)	50 (3)
Cyclophosphamide and rituximab	0(0)	0 (0)	6.7 (1)	0 (0)	0 (0)	0 (0)
Azathioprine	20 (3)	60 (9)	6.7 (1)	73.3 (11)	0 (0)	33.3 (2)
Corticosteroids alone	0 (0)	26.7 (4)	0 (0)	13.3 (2)	0 (0)	0 (0)
Other (MMF)	0 (0)	6.7 (1)	0 (0)	0 (0)	0 (0)	0 (0)
No treatment	0 (0)	0 (0)	0 (0)	0 (0)	0 (0)	16.7 (1)

**Table 5:** Summary of progression to ESRD/death, CKD, and preservation of renal function: induction and maintenance treatment.

Outcome	Induction: cyclophosphamide (*n* = 24)	Induction: rituximab (*n* = 7)	Induction: cyclophosphamide and rituximab (*n* = 1)	Induction: azathioprine (*n* = 22)	Maintenance: rituximab (*n* = 6)	Maintenance: azathioprine (*n* = 4)	Maintenance: corticosteroids alone (*n* = 6)	Maintenance: MMF (*n* = 1)	Maintenance: no treatment (*n* = 1)
ESRD or death, % (n)	41.7 (10)	28.6 (2)	0 (0)	75 (3)	6.7 (1)	40.9 (9)	66.7 (4)	100 (1)	0 (0)
CKD, % (n)	45.8 (11)	28.6 (2)	100 (1)	25 (1)	33.3 (2)	50 (11)	33.3 (2)	0 (0)	0 (0)
Preservation of renal function, % (*n*)	12.5 (3)	42.9 (3)	0 (0)	0 (0)	50 (3)	9.1 (2)	0 (0)	0 (0)	100 (1)

## Data Availability

The data and materials used and/or analysed during the current study are available from the corresponding author on reasonable request.

## Supplementary Materials

Supplementary Materials Reporting guidleines STROBE_checklist.
